# Studying Disease Reinfection Rates, Vaccine Efficacy, and the Timing of Vaccine Rollout in the Context of Infectious Diseases: A COVID-19 Case Study

**DOI:** 10.3390/ijerph22050731

**Published:** 2025-05-03

**Authors:** Elizabeth B. Amona, Indranil Sahoo, Edward L. Boone, Ryad Ghanam

**Affiliations:** 1 Department of Statistical Sciences and Operations Research, Virginia Commonwealth University, Richmond, VA 23284, USA; amonaeb@vcu.edu (E.B.A.); elboone@vcu.edu (E.L.B.); 2 Department of Liberal Arts and Sciences, Virginia Commonwealth University in Qatar, Education City, Doha P.O. Box 8095, Qatar; raghanam@vcu.edu

**Keywords:** Bayes factor, compartmental models, COVID-19, epidemiology, Hellinger distance, kernel density estimation

## Abstract

The COVID-19 pandemic has highlighted the intricate nature of disease dynamics, extending beyond transmission patterns to the complex interplay of intervention strategies. In the post-COVID-19 era, reinfection has emerged as a critical factor, shaping how we model disease progression, evaluate immunity, and assess the effectiveness of public health interventions. This research uniquely explores the varied efficacy of existing vaccines and the pivotal role of vaccination timing in the context of COVID-19. Departing from conventional modeling, we introduce two models that account for the impact of vaccines on infections, reinfections, and deaths. We estimate model parameters under the Bayesian framework, specifically utilizing the Metropolis–Hastings Sampler. We conduct data-driven scenario analyses for the State of Qatar, quantifying the potential duration during which the healthcare system could have been overwhelmed by an influx of new COVID-19 cases surpassing available hospital beds. Additionally, the research explores similarities in predictive probability distributions of cumulative infections, reinfections, and deaths, employing the Hellinger distance metric. Comparative analysis, utilizing the Bayes factor, underscores the plausibility of a model assuming a different susceptibility rate to reinfection, as opposed to assuming the same susceptibility rate for both infections and reinfections. Results highlight the adverse outcomes associated with delayed vaccination, emphasizing the efficacy of early vaccination in reducing infections, reinfections, and deaths. Our research advocates for prioritization of early vaccination as a key strategy in effectively combating future pandemics, thereby providing vital insights for evidence-based public health interventions.

## 1. Introduction

The relentless battle against severe acute respiratory syndrome coronavirus 2 (SARS-CoV-2) has unveiled new complexities in the dynamics of infectious diseases and the effectiveness of interventions. In the post-pandemic era, one aspect that has garnered significant attention is the phenomenon of reinfection—a topic that is both timely and imperative for further exploration. Reinfection with SARS-CoV-2 occurs when an individual contracts the virus, recovers, and subsequently becomes infected again. While most reinfections tend to be mild, severe illness can also occur [1,2]. People who are reinfected can also spread the virus to others, and staying up to date with vaccine doses and starting treatment within days after developing symptoms can decrease a person’s risk of experiencing severe illness from reinfection [2].

The State of Qatar, like many countries, has experienced distinct waves of COVID-19 infections. From the initial surge of cases to the subsequent introduction of new variants, Qatar’s experience serves as an insightful case study. In Qatar, an initial wave of infections occurred between March and June 2020, resulting in the development of detectable antibodies against SARS-CoV-2 in approximately 40% of the population. Subsequently, the country experienced two consecutive waves of infections from January to May 2021, triggered by the emergence of the B.1.1.7 (Alpha) and B.1.351 (Beta) variants [3]. Other variants, such as Omicron and its subvariants, have also emerged [4]. Understanding the patterns of infections, reinfections, and associated mortality in the presence of effective vaccination is crucial to guide effective public health strategies. This research offers a comprehensive methodology to assess the impact of variations in vaccine efficacy and timing on infectious disease dynamics. The proposed methodology was implemented to analyze the dynamics of COVID-19 in terms of infections, reinfections, and deaths within Qatar.

Various studies have investigated the impact of vaccination effectiveness on infections and deaths globally, employing mathematical frameworks or case–control designs. Ref. [5] developed the SEIRDV model (susceptible, exposed, infected, recovered, deaths, and vaccinated) to study the effectiveness of vaccination in in reducing secondary cases and the mortality rate in Qatar. The authors concluded that vaccines drastically reduced the basic reproduction number ($R_{0}$) and saved lives. Ref. [6] employed an SEIR model (susceptible, exposed, infected, and recovered) to determine the required vaccine efficiency to diminish infection and mortality peaks in Italy, India, and Australia. Results indicated substantial reductions with specific vaccine efficiency and coverage combinations; for example, a 75% efficient vaccine administered to 50% of the population can reduce the peak number of infected individuals by nearly 50% in Italy. Ref. [7] conducted a test–negative case–control study in England to estimate vaccine effectiveness against Omicron and Delta variants, revealing effectiveness against symptomatic disease but highlighting waning protection over time. Some studies have further delved into the possibility of reinfection after the waning of vaccines. Ref. [8] performed a retrospective cohort study in Israel, revealing a sixfold higher reinfection rate among the unvaccinated compared to the vaccinated, highlighting vaccine effectiveness and waning protection. Additional cohort studies on vaccine effectiveness and reinfection risks post waning have been documented in [9,10,11,12,13,14]. Notably, these studies exclusively employed cohort designs. Ref. [15] explored scenario analysis early in the pandemic using an SEIR model in South Africa in a different context.

More recently, ref. [16,17] investigated the effects of natural immunity on reinfection over time, with a particular focus on the emergence of the Omicron variant. Romero-Ibarguengoitia et al. [18] analyzed the relationship between reinfections and the risk of long COVID through an observational cross-sectional study. Additionally, ref. [19] developed mathematical models to examine how different vaccine types influence immune response over time. While this work provided insights into vaccine-induced immunity, it did not explore reinfection dynamics or the distinction between post-infection and vaccine-derived immunity, which are key aspects of our study.

Thus, after an extensive literature review, several research gaps in the context of studying the dynamics of infectious diseases emerged. Firstly, we think that timely vaccine availability is a significant concern that requires further investigation. Secondly, several mathematical models have been recently developed to study COVID-19 reinfection. Notable examples include [20,21,22]. However, there is a notable absence of mathematical models that explicitly explain the degree of susceptibility after infection resulting from the waning of vaccination. The lack of such models leaves a critical gap in understanding the dynamics of post-vaccination susceptibility. Thirdly, a gap also exists in the realm of scenario analysis on vaccine efficacy. No study has comprehensively explored how variations in vaccine efficacy might impact infection, reinfection, and mortality rates. This gap underscores the need for research that delves into the broader implications of vaccine efficacy for different aspects of disease dynamics.

The uniqueness of our study lies in its emphasis on scenario analysis of vaccine efficacy, closely reflecting the real-world dynamics of vaccination efforts. In this context, we explore the impact of these variations on the spread of the virus, as well as the occurrence of reinfections and mortality. In conjunction with vaccine efficacy, the timing (early or late) of vaccine rollout is identified as an important factor. The choice between early and late vaccine administration has implications for achieving population-level immunity and attenuating the impact of the virus. To better reflect real-world scenarios, we propose two mathematical models with different dynamics under different assumptions (see Section 2). This work deviates from traditional mathematical modeling by not only estimating parameters and studying system dynamics based on real data but also projecting various scenarios relevant to infectious diseases, specifically COVID-19. Furthermore, while conventional models change parameter values to study the efficacy and impact of vaccination, we changed when the interventions and/or vaccinations were implemented to explore their dynamics, which makes our study novel. Given our proposed models and the estimated parameter values, we seek to address the following research questions: What would have happened if vaccines had been administered at different time points—either earlier or later? In scenarios with limited government interventions, what potential consequences might arise? Is there a possibility that delaying vaccine administration could lead to a scenario where the number of infections exceeds the capacity of the country’s healthcare system, specifically in terms of available hospital beds? We aim to calculate the number of days on which such system breakdowns might occur should vaccines be administered late. Our objective is to apply the proposed mathematical models to explore the consequences of different vaccination strategies and their impact on reinfection dynamics and healthcare capacity, moving beyond parameter estimation to understand real-world implications.

### Motivation for Model Comparison

In order to explain the real-world dynamics of infectious diseases, we introduce two mathematical models, each offering a different viewpoint on the complex interaction among diseases, immunity, and vaccination. The first model assumes that individuals who recover from their initial infection might exhibit different susceptibility rates upon re-exposure. This assumption reflects the idea that immunity wanes over time and that those who have recovered from the disease may still be at risk, although at a different rate than before reinfection. Thus, this model considers different susceptibility rates among different segments of the population and their impact on the overall course of the disease.

In contrast, the second model does not account for the varying susceptibility of those who have recovered, thereby taking a more straightforward approach and assuming a uniform susceptibility rate among all individuals. We describe the two models in Section 2, exploring the following question: Does the first model, with its complex assumptions about variable susceptibility, represent real-world dynamics better or does the simplicity of the second model provide a more accurate picture? Thus, our approach provides data-driven insights into the real-world implications of varying susceptibility rates, waning immunity, and vaccination efficacy. As such, this study provides a clearer understanding of disease dynamics in the presence of evolving immunity and guidance for public health interventions and decision-making in a complex, ever-changing world.

## 2. The Compartmental Models

In this section, we present two models reflecting the practical implications of government policies introduced during the pandemic. To provide clarity on our modeling approach, we enumerate some key assumptions about these models:

1. Emigration and birth are excluded due to the closure of the international airport and a blockade during the study period, preventing immigration and emigration. Those entering during this time underwent a 10-day quarantine.
2. Individuals in the infected (symptomatic) compartment are quarantined, minimizing interactions with the susceptible population, except for caregivers who contract the disease separately.
3. Vaccination is targeted towards exposed individuals who recover, while those who have already been infected and recovered do not receive the vaccine, having developed antibodies.
4. Susceptible ($S_{1}$ and *S*), exposed, and recovered individuals are vaccinated at the same rate.

Since all of these model assumptions were reasonably valid during the time of data collection, Qatar is an ideal case study for this work. In these models, *t* serves as a time index, denoting the number of days elapsed since the first recorded case of COVID-19 within the population of interest. At any given time (*t*), S(t) denotes the total number of susceptible individuals, $S_{1}\left(t\right)$ represents the number of individuals who are susceptible and have not contracted the disease, E(t) denotes the number of individuals who are in the exposed state, I(t) denotes the total number of individuals who are infected (displaying symptoms), $R_{E}\left(t\right)$ denotes the cumulative number of individuals who have recovered following exposure, $R_{I}\left(t\right)$ denotes the cumulative number of individuals who have recovered following infection, D(t) denotes the cumulative number of deaths, $S_{2}\left(t\right)$ denotes the cumulative number of individuals who have regained susceptibility after their initial infection, $I_{I}\left(t\right)$ denotes the cumulative number of individuals who have experienced reinfection, $R_{R}\left(t\right)$ denotes the cumulative number of individuals who have recovered following reinfection, and V(t) denotes the cumulative number of vaccinated individuals.

### 2.1. The $S_{1}$EIRDV$S_{2}I_{I}R_{R}$ Model

**Figure 1:**
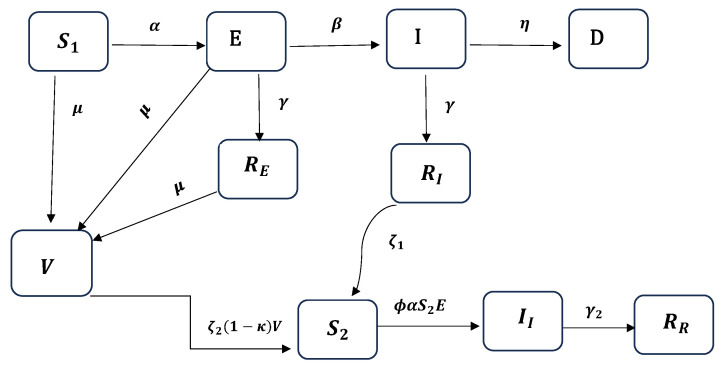
Schematic diagram of the $S_{1}$EIRDV$S_{2}I_{I}R_{R}$ model for COVID-19.

In addition to the inherent model assumptions, we assume that individuals who have previously been infected develop antibodies for a certain period before these antibodies naturally wane. Subsequently, these individuals transition to a different susceptible compartment ($S_{2}$) at a different rate compared to the initial susceptible compartment ($S_{1}$). Additionally, we assume that the waning of natural immunity and immunity resulting from vaccination occur at different rates, denoted as $\zeta_{1}$ (representing natural immunity waning) and $\zeta_{2}\left(1-\kappa \right)$ (representing vaccination-induced waning), respectively. Moreover, we assume that all individuals who experience reinfection recover and transition to a secondary recovery compartment ($R_{R}$). We do not consider any deaths following reinfection. This assumption is supported by research indicating that the risk of severe reinfection in the State of Qatar was only approximately 1% of the risk faced by individuals who had not been previously infected [3]. Thus, the $S_{1}$EIRDV$S_{2}I_{I}R_{R}$ compartmental model comprises ten distinct compartments, a visual representation of which is presented in Figure 1.

Following the flow diagram presented in Figure 1 and the modeling assumptions described above, Model 1 can be formally represented by the following system of equations:

(1) $$\begin{array}{ccc} \frac{dS_{1}}{dt} & = & -\alpha S_{1}\left(t\right)E\left(t\right)-\mu S_{1}\left(t\right),\\ \frac{dE}{dt} & = & \alpha S_{1}\left(t\right)E\left(t\right)-\left(\beta +\gamma_{1}+\mu \right)E\left(t\right),\\ \frac{dI}{dt} & = & \beta E\left(t\right)-\left(\gamma_{1}+\eta \right)I\left(t\right),\\ \frac{dR_{E}}{dt} & = & \gamma_{1}E\left(t\right)-\mu R_{E}\left(t\right),\\ \frac{dR_{I}}{dt} & = & \gamma_{1}I\left(t\right)-\zeta_{1}R_{I}\left(t\right),\\ \frac{dD}{dt} & = & \eta I(t),\\ \frac{dS_{2}}{dt} & = & \zeta_{1}R_{I}\left(t\right)-\phi \alpha S_{2}\left(t\right)E\left(t\right)+\zeta_{2}\left(1-\kappa \right)V\left(t\right),\\ \frac{dI_{I}}{dt} & = & \phi \alpha S_{2}\left(t\right)E\left(t\right)-\gamma_{2}I_{I}\left(t\right),\\ \frac{dR_{R}}{dt} & = & \gamma_{2}I_{I}\left(t\right),\\ \frac{dV}{dt} & = & \mu \left(S_{1}\left(t\right)+E\left(t\right)+R_{E}\left(t\right)\right)-\zeta_{2}\left(1-\kappa \right)V\left(t\right), \end{array}$$

with the following constraints: $\;S_{1}\left(t\right)\geq 0,\;S_{2}\left(t\right)\geq 0,\;E\left(t\right)\geq 0,\;I\left(t\right)\geq 0,\;I_{I}\left(t\right)\geq 0,\;R_{E}\left(t\right)\geq 0,\;R_{I}\left(t\right)\geq 0,\;R_{R}\left(t\right)\geq 0,\;D\left(t\right)\geq 0,\;\mathrm{and}\;V\left(t\right)\geq 0$. All parameters in system (1) are positive and are explained as follows: α is the transmission rate (per day × individual^2^) from susceptible to exposed; β denotes the rate (per day) at which exposed individuals become infected (symptomatic); $\gamma_{1}$ is the rate (per day) at which infected individualsbecome recovered; ϕ is the rate (per day × individual^2^) at which those who have recovered from a first infection become reinfected; $\gamma_{2}$ is the rate (per day) at which reinfected individualsbecome recovered; the vaccination rate (per day) is denoted by μ; the efficacy of the vaccine is denoted by κ, where 0<κ≤1; the waning of natural immunity and vaccine immunity is denoted by $\zeta_{1}$ and $\zeta_{2}$, respectively; and the mortality rate (per day) for infected individuals is denoted by η. Note that $\gamma_{2}=1$, which means that everyone becomes reinfected recovers after a while as a consequence of one of our assumptions.

### 2.2. The SEIRDV$I_{I}R_{R}$ Model

**Figure 2:**
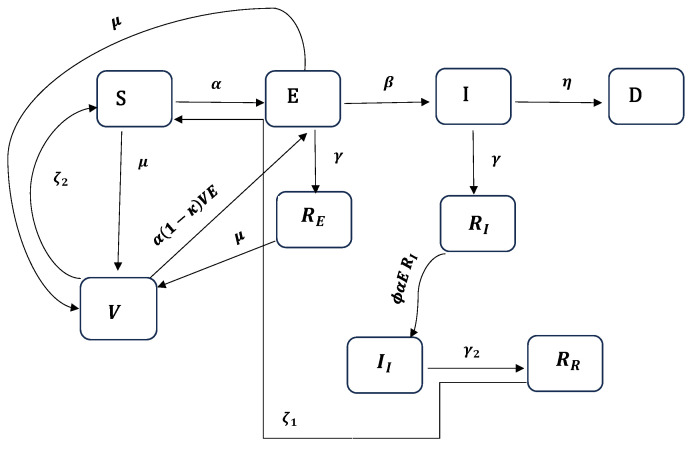
Schematic diagram of the SEIRDV$I_{I}R_{R}$ model for COVID-19.

In this model, we assume that individuals who recover after the first infection could become reinfected within a short period of time if they come in contact with exposed individuals. Here, we do not have a second susceptible compartment ($S_{2}$) as we had in Model (1). This means that when an individual recovers, they automatically move into the reinfected compartment after natural immunity wanes, as opposed to Model (1), which assumes that there is a delay between recovery and reinfection through the second susceptible compartment ($S_{2}$) and that the susceptibility rate differs. The remaining assumptions of this model are similar to those of the previous model. A visual representation is presented in Figure 2.

Following the flow diagram presented in Figure 2 and the modeling assumptions described above, we represent Model (2) using the following system of equations:

(2) $$\begin{array}{ccc} \frac{dS}{dt} & = & -\alpha S\left(t\right)E\left(t\right)-\mu S\left(t\right)+\zeta_{1}R_{R}\left(t\right)+\zeta_{2}V\left(t\right),\\ \frac{dE}{dt} & = & \alpha S\left(t\right)E\left(t\right)-\left(\beta +\gamma_{1}+\mu \right)E\left(t\right)+\alpha \left(1-\kappa \right)V\left(t\right)E\left(t\right),\\ \frac{dI}{dt} & = & \beta E\left(t\right)-\left(\gamma_{1}+\eta \right)I\left(t\right),\\ \frac{dR_{E}}{dt} & = & \gamma_{1}E\left(t\right)-\mu R_{E}\left(t\right),\\ \frac{dR_{I}}{dt} & = & \gamma_{1}I\left(t\right)-\alpha \phi E\left(t\right)R_{I}\left(t\right),\\ \frac{dD}{dt} & = & \eta I(t),\\ \frac{dI_{I}}{dt} & = & \alpha \phi E\left(t\right)R_{I}\left(t\right)-\gamma_{2}I_{I}\left(t\right),\\ \frac{dR_{R}}{dt} & = & \gamma_{2}I_{I}\left(t\right)-\zeta_{1}R_{R}\left(t\right),\\ \frac{dV}{dt} & = & \mu \left(S\left(t\right)+E\left(t\right)+R_{E}\left(t\right)\right)-\zeta_{2}V\left(t\right)-\alpha \left(1-\kappa \right)V\left(t\right)E\left(t\right), \end{array}$$

with the following constraints: $\;S\left(t\right)\geq 0,\;E\left(t\right)\geq 0,\;I\left(t\right)\geq 0,\;I_{I}\left(t\right)\geq 0,\;R_{E}\left(t\right)\geq 0,\;R_{I}\left(t\right)\geq 0,\;R_{R}\left(t\right)\geq 0,\;D\left(t\right)\geq 0,\;\mathrm{and}\;V\left(t\right)\geq 0$. All model parameters can be interpreted similarly to those discussed in Section 2.1.

## 3. Statistical Methodology for Model Inference and Model Comparison

### 3.1. The Bayesian Analysis Framework

Given the evolving nature of the pandemic and interventions by governments to influence various parameters, assuming a “steady state” for the dynamic system is inappropriate. Hence, we allow the transmission, recovery, and reinfection rates in our models to vary over time. We define the transmission rate vector as $\alpha ={\left(\alpha_{0},\alpha_{1},...\alpha_{m}\right)}^{T}$, where $\alpha \left(t\right)=\alpha_{m-1}\;\mathrm{if}\;t_{m-1}\leq t<t_{m}$. Since α(t)>0 for all *t*, we have $\alpha_{i}>0,i=0,1,\ldots ,m$. Similarly, we define the recovery rate vector as $\gamma_{1}={\left(\gamma_{10},\gamma_{11},\cdots ,\gamma_{1l}\right)}^{T},l\leq m$, where each γ is independent, denoting the changed recovery rate once an intervention has been administered. We write $\gamma_{1}\left(t\right)=\gamma_{1\;l-1},\;\mathrm{if}\;t_{l-1}^{\prime }\leq t<t_{l}^{\prime }$, where $t_{1}^{\prime },t_{2}^{\prime },\ldots ,t_{l}^{\prime }\in \left\{t_{1},t_{1}+1,t_{1}+2,\ldots ,t_{2},t_{2}+1,\ldots ,t_{m}\right\}$. Since $\gamma_{1}\left(t\right)>0$ for all *t*, we have $\gamma_{1j}>0,j=0,1,\ldots ,l$. Similarly, the reinfection rate vector is written as $\phi ={\left(\phi_{0},\phi_{1},...\phi_{p}\right)}^{T}$, where $\phi \left(t\right)=\phi_{p-1}\;\mathrm{if}\;t_{p-1}^{\prime \prime }\leq t<t_{p}^{\prime \prime }$ with $\phi_{k}>0,k=0,1,\ldots ,p$ and $t_{1}^{\prime \prime },t_{2}^{\prime \prime },\ldots ,t_{p}^{\prime \prime }\in \left\{t_{1},t_{1}+1,t_{1}+2,\ldots ,t_{2},t_{2}+1,\ldots ,t_{m}\right\}$. Here, $t^{\prime }$ and $t^{\prime \prime }$ denote time stamps at which the recovery and reinfection rates change, respectively.

Thus, for i=0,1,…,m,j=0,1,…,l and k=0,1,…,p, the system of equations in (1) becomes

(3) $$\begin{array}{ccc} \frac{d\lambda_{S_{1}}}{dt} & = & -\alpha_{i}\lambda_{S_{1}}\left(t\right)\lambda_{E}\left(t\right)-\mu \lambda_{S_{1}}\left(t\right),\\ \frac{d\lambda_{E}}{dt} & = & \alpha_{i}\lambda_{S_{1}}\left(t\right)\lambda_{E}\left(t\right)-\left(\beta +\gamma_{1j}+\mu \right)\lambda_{E}\left(t\right),\\ \frac{d\lambda_{I}}{dt} & = & \beta \lambda_{E}\left(t\right)-\left(\gamma_{1j}+\eta \right)\lambda_{I}\left(t\right),\\ \frac{d\lambda R_{E}}{dt} & = & \gamma_{1j}\lambda_{E}\left(t\right)-\mu \lambda_{R_{E}}\left(t\right),\\ \frac{d\lambda_{R_{I}}}{dt} & = & \gamma_{1j}\lambda_{I}\left(t\right)-\zeta_{1}\lambda_{R_{I}}\left(t\right),\\ \frac{d\lambda_{D}}{dt} & = & \eta \lambda_{I}\left(t\right),\\ \frac{d\lambda_{S_{2}}}{dt} & = & \zeta_{1}\lambda_{R_{I}}\left(t\right)-\phi_{k}\alpha_{i}\lambda_{S_{2}}\left(t\right)\lambda_{E}\left(t\right)+\zeta_{2}\left(1-\kappa \right)\lambda_{V}\left(t\right),\\ \frac{d\lambda_{I_{I}}}{dt} & = & \phi_{k}\alpha_{i}\lambda_{S_{2}}\left(t\right)\lambda_{E}\left(t\right)-\gamma_{2}\lambda_{I_{I}}\left(t\right),\\ \frac{d\lambda_{R_{R}}}{dt} & = & \gamma_{2}\lambda_{I_{I}}\left(t\right),\\ \frac{d\lambda_{V}}{dt} & = & \mu \left(\lambda_{S_{1}}\left(t\right)+\lambda_{E}\left(t\right)+\lambda_{R_{E}}\left(t\right)\right)-\zeta_{2}\left(1-\kappa \right)\lambda_{V}\left(t\right), \end{array}$$

Similarly, the system of equations in (2) becomes

(4) $$\begin{array}{ccc} \frac{d\lambda_{S_{1}}}{dt} & = & -\alpha_{i}\lambda_{S_{1}}\left(t\right)\lambda_{E}\left(t\right)-\mu \lambda_{S_{1}}\left(t\right)+\zeta_{1}\lambda_{R_{R}}\left(t\right)+\zeta_{2}\lambda_{V}\left(t\right),\\ \frac{d\lambda_{E}}{dt} & = & \alpha_{i}\lambda_{S_{1}}\left(t\right)\lambda_{E}\left(t\right)-\left(\beta +\gamma_{1j}+\mu \right)\lambda_{E}\left(t\right)+\alpha_{i}\left(1-\kappa \right)\lambda_{V}\left(t\right)\lambda_{E}\left(t\right),\\ \frac{d\lambda_{I}}{dt} & = & \beta \lambda_{E}\left(t\right)-\left(\gamma_{1j}+\eta \right)\lambda_{I}\left(t\right),\\ \frac{d\lambda R_{E}}{dt} & = & \gamma_{1j}\lambda_{E}\left(t\right)-\mu \lambda_{R_{E}}\left(t\right),\\ \frac{d\lambda_{R_{I}}}{dt} & = & \gamma_{1j}\lambda_{I}\left(t\right)-\alpha_{i}\phi_{k}\lambda_{E}\left(t\right)\lambda_{R_{I}}\left(t\right),\\ \frac{d\lambda_{D}}{dt} & = & \eta \lambda_{I}\left(t\right),\\ \frac{d\lambda_{I_{I}}}{dt} & = & \alpha_{i}\phi_{k}\lambda_{E}\left(t\right)\lambda_{R_{I}}\left(t\right)-\gamma_{2}\lambda_{I_{I}}\left(t\right),\\ \frac{d\lambda_{R_{R}}}{dt} & = & \gamma_{2}\lambda_{I_{I}}\left(t\right)-\zeta_{1}\lambda_{R_{R}}\left(t\right),\\ \frac{d\lambda_{V}}{dt} & = & \mu \left(\lambda_{S}\left(t\right)+\lambda_{E}\left(t\right)+\lambda_{R_{E}}\left(t\right)\right)-\zeta_{2}\lambda_{V}\left(t\right)-\alpha_{i}\left(1-\kappa \right)\lambda_{V}\left(t\right)\lambda_{E}\left(t\right), \end{array}$$

where $\lambda_{S_{1}}\left(t\right),\lambda_{E}\left(t\right),\lambda_{I}\left(t\right),\lambda_{R_{E}}\left(t\right),\lambda_{R_{I}},\lambda_{S_{2}}\left(t\right),\lambda_{V}\left(t\right),\lambda_{I_{I}}\left(t\right),\lambda_{R_{R}}\left(t\right)$, and $\lambda_{D}\left(t\right)$ denote the mean parameters. The prior distributions of the parameters in Models (3) and (4) are chosen as follows:

(5) $$\begin{array}{cc} \alpha_{i} & ∼Exp(1),\;i=0,1,\ldots ,m=10,\\ \beta & ∼Exp(1),\\ \gamma_{1j} & ∼Exp(1),\;j=0,1,\ldots ,l=3,\\ \zeta_{1} & ∼Exp(1),\\ \zeta_{2} & ∼Exp(1),\\ \mu & ∼Exp(1),\\ \phi_{k} & ∼Exp(1),\;k=0,1,\ldots ,p=3,\\ \gamma_{2} & ∼Exp(1),\\ \eta & ∼Exp(1),\\ \kappa & ∼Be(1,1), \end{array}$$

The likelihoods for I(t), $R_{I}\left(t\right)$, $I_{I}\left(t\right)$, $R_{R}\left(t\right)$, D(t), and V(t) in Models (3) and (4) are given by

(6) $$\begin{array}{cc} I(t) & ∼Poisson\left(\lambda_{I}\left(t\right)\right),\\ R_{I}\left(t\right) & ∼Poisson\left(\lambda_{R_{I}}\left(t\right)\right),\\ D(t) & ∼Poisson\left(\lambda_{D}\left(t\right)\right),\\ I_{I}\left(t\right) & ∼Poisson\left(\lambda_{I_{I}}\left(t\right)\right),\\ R_{R}\left(t\right) & ∼Poisson\left(\lambda_{R_{R}}\left(t\right)\right),\\ V(t) & ∼Poisson\left(\lambda_{V}\left(t\right)\right). \end{array}$$

Similar prior and likelihood assumptions were established in [5]. Note that $S_{1}\left(t\right)$, $S_{2}\left(t\right)$, and E(t) in Model (3) and S(t), E(t) in model (4) are not included in the likelihood, as they are latent states, meaning they are not directly observed. Additionally, we did not explicitly divide the exposed compartment into asymptomatic and presymptomatic subcategories because our analysis is informed by available data, and such data are not present. To avoid introducing redundant compartments, we opted to simplify our model by embedding all these categories within the exposed compartment, particularly since they are treated as latent in the analysis, meaning that even if modeled explicitly, they would still remain unobserved. While the true joint likelihood for $\left\{S_{1}\left(t\right),S_{2}\left(t\right),E\left(t\right),I\left(t\right),R_{E}\left(t\right),R_{I}\left(t\right),I_{I}\left(t\right),R_{R}\left(t\right),V\left(t\right),D\left(t\right)\right\}$ should be multinomial, with three latent states in (3) and two latent states in (4), one of which is the largest state, the multinomial approach is challenging to apply. Hence, this work adopts Poisson likelihoods as an approximation. The posterior distribution for both models can be calculated based on the prior and likelihood specifications mentioned above. Since solving the posterior distribution analytically is difficult, we employ the Markov Chain Monte Carlo (MCMC) technique to sample from the posterior distribution [23]. Specifically, we utilized the Metropolis–Hastings sampler [5,24,25].

To tune the sampler, a series of short chains was generated and analyzed for convergence and adequate acceptance rates. These initial short chains were discarded as “burn-in” samples. The tuned sampler was used to generate 50,000 samples from the posterior distribution, and trace plots were visually examined to ensure convergence (not included). All inferences, including parameter estimation and uncertainty quantification, were made from these 50,000 posterior draws. The model and sampling algorithm were custom-programmed in R statistical programming language version 3.6.3. The computations take approximately 10,800 s using an AMD A10-9700 3.50 GHz processor with 16 GB of RAM to obtain 50,000 draws from the posterior distribution. For more details on statistical inference, see [26,27,28].

### 3.2. Bayesian Model Comparison Using Bayes Factor

In Bayesian model comparison, the Bayes Factor (BF) is a robust and versatile tool that helps researchers evaluate the relative probability of different models. By considering both parameter uncertainty and prior information, it offers a comprehensive and principled approach to model selection. When working with posterior samples obtained through methods like the Metropolis–Hastings sampler, the Bayes factor can be especially valuable for assessing the probability of competing models and informing decision making in a wide range of scientific disciplines.

Furthermore, the Bayes factor quantifies the support for one statistical model (Model 1) over another (Model 2) given the observed data, that is, the Bayes factor offers a formal framework to test the following hypothesis based on the principles of Bayesian probability theory [29].

$$\begin{array}{cc} \mathrm{Null}\;\mathrm{Hypothesis}(H_{0}) & :\mathrm{Model}\;1\;\mathrm{is}\;\mathrm{more}\;\mathrm{probable}\;\mathrm{than}\;\mathrm{Model}\;2.\;\\ \mathrm{Alternative}\;\mathrm{Hypothesis}(H_{A}) & :\mathrm{Model}\;2\;\mathrm{is}\;\mathrm{more}\;\mathrm{probable}\;\mathrm{than}\;\mathrm{Model}\;1. \end{array}$$

The Bayes factor is computed as the ratio of the marginal likelihoods of the two models:

$$BF_{12}=\frac{P(Data|Model\;1)}{P(Data|Model\;2)},$$

where P(Data|Model1) and P(Data|Model2) are the probability densities of Data under Model 1 and Model 2, respectively. A Bayes factor greater than 1 indicates that Model 1 is favored over Model 2. The magnitude of $BF_{12}$ provides a measure of the strength of this preference. Larger $BF_{12}$ values indicate stronger evidence in favor of Model 1. Conversely, a Bayes factor of less than 1 suggests that Model 2 is favored over Model 1. A Bayes factor close to 1 suggests that the two models are equally plausible based on the data, thereby providing no strong evidence in favor of one model over the other [30,31]. In our work, we compare the two models using the Bayes factor to provide a data-driven assessment of which model better represents the dynamics of the pandemic in the State of Qatar over our study period of 28 February 2020–29 August 2021.

### 3.3. Assessing ‘Closeness’ of Density Plots Using Hellinger Distance

We created density plots to visually compare posterior predictive distributions (PPDs) of cumulative infected, cumulative reinfected, and cumulative deaths across various scenarios. Additionally, we quantitatively assessed the ‘closeness’ between two density plots, enhancing the robustness of our statistical analysis. The Hellinger distance is a metric for assessing the similarity of density plots. This metric is useful for quantifying the separation or overlap between probability density functions, among other characteristics, thereby offering a robust and objective approach to evaluating the ‘closeness’ between posterior distributions. While Kullback–Leibler divergence is usually a more popular choice, it is not symmetric [32] and, hence, is not suitable for our purposes. The Hellinger distance is defined as

$$H^{2}\left(f,g\right)=\frac{1}{2}\int {\left(\sqrt{f(x)}-\sqrt{g(x)}\right)}^{2}dx=1-\int \sqrt{f(x)g(x)}dx,\;\;\;0\leq H^{2}\left(f,g\right)\leq 1.$$

where f(x) and g(x) are the two probability density functions being compared. The integral is computed over the entire support of the distributions [33,34]. The Hellinger distance produces a value between 0 and 1, where $H^{2}=0$ indicates that the two density plots are identical, $0<H^{2}<1$ implies that the density plots share some degree of similarity, and $H^{2}=1$ suggests that the density plots are completely dissimilar with no overlap [35].

In our case, the cumulative posterior predictive distributions (f(x) and g(x)) for all scenarios are estimated using kernel density smoothing (KDE) with a Gaussian kernel and default bandwidth as suggested by [36]. Smoothing is implemented using the R density() function from the stats package. Since KDEs can extend beyond the original data range, while comparing two KDEs, we ensure they (i) cover a common range of values and (ii) discretize that range identically, using an equally spaced set of points in the analysis.

## 4. Results

**Figure 3:**
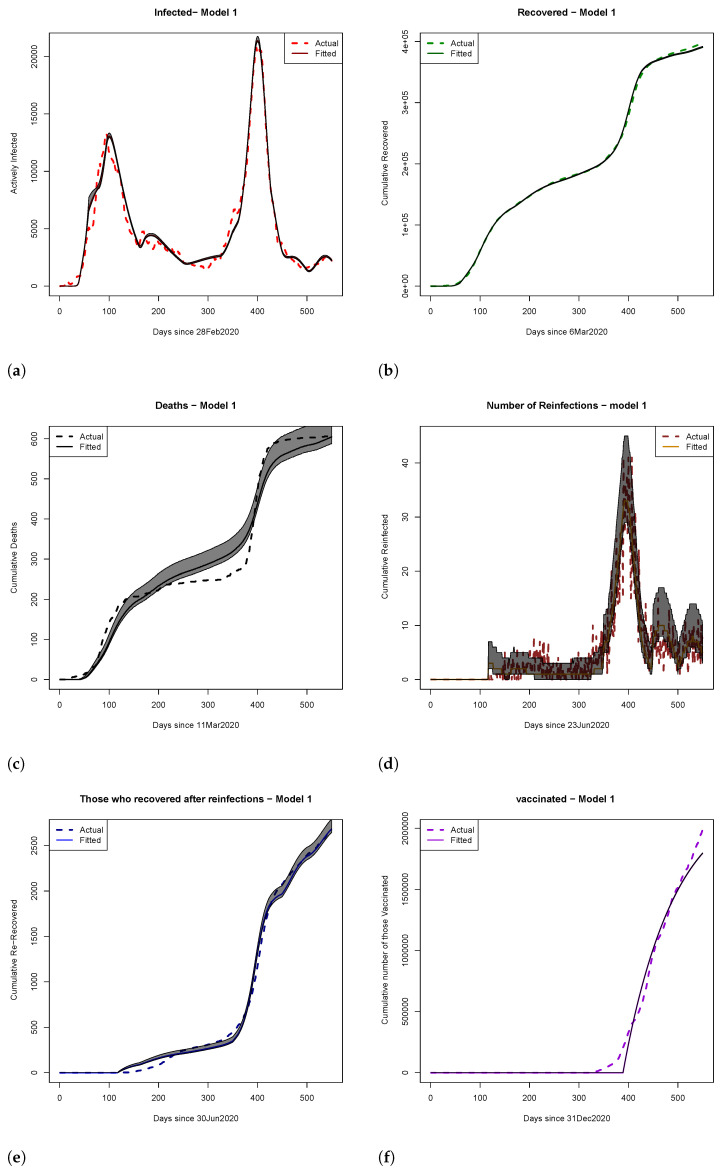
Plots of (**a**) active infections, (**b**) cumulative recovered, (**c**) cumulative deaths, (**d**) number of reinfections, (**e**) cumulative recovered after reinfections, and (**f**) cumulative number of vaccinated for Model 1. The estimated quantiles (0.025, 0.5, and 0.975) were calculated using 50,000 samples from the posterior distribution. The shaded regions represent 95% credible Bayesian intervals (i.e., the range between the 2.5th and 97.5th percentiles). Solid lines indicate fitted values from Model 1, and dotted lines show observed data.

**Figure 4:**
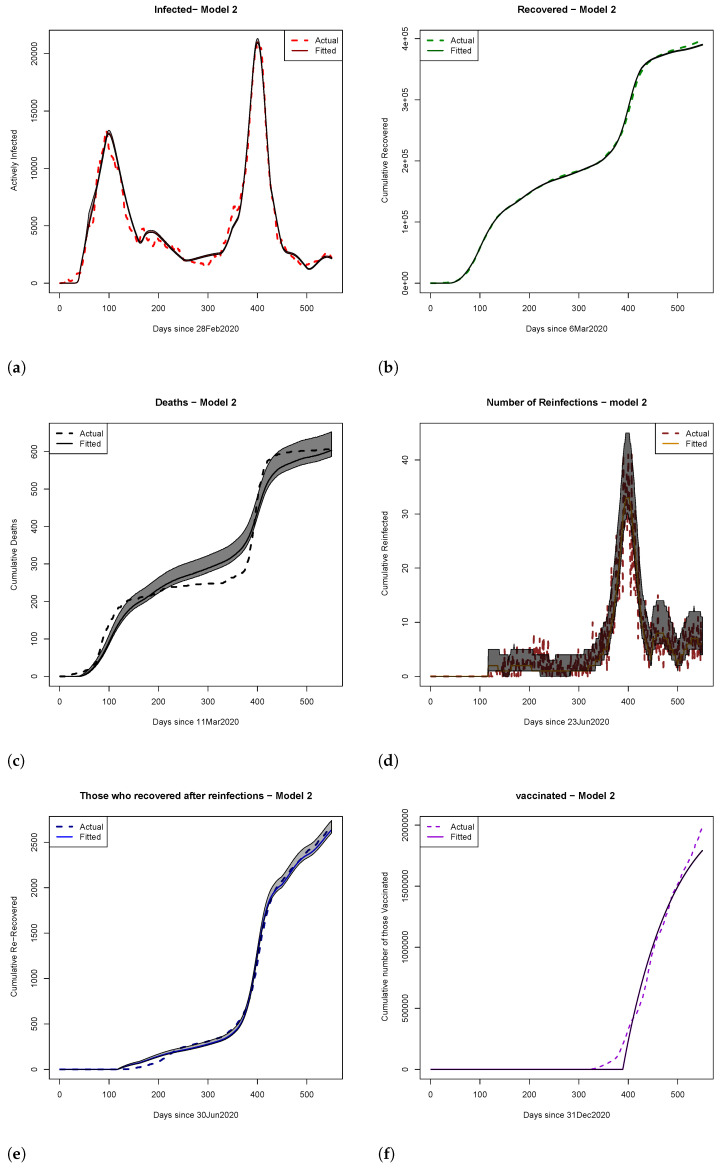
Plots of (**a**) active infections, (**b**) cumulative recovered, (**c**) cumulative deaths, (**d**) number of reinfections, (**e**) cumulative recovered after reinfections, and (**f**) cumulative number of vaccinated for Model 2. The estimated quantiles (0.025, 0.5, and 0.975) were calculated using 50,000 samples from the posterior distribution. The shaded regions represent 95% credible Bayesian intervals (i.e., the range between the 2.5th and 97.5th percentiles). Solid lines indicate fitted values from Model 2, and dotted lines show observed data.

In this section, we assess which of the two introduced mathematical models better captures the real-world dynamics of the COVID-19 pandemic in the State of Qatar. The initial conditions used for our analysis are presented in Table 1. We also provide the results of fitting the two models, including the estimated mean parameter, 2.5 and 97.5 percentiles, and pseudo-$R^{2}$ of the fitted models (shown in Table 2). The results were obtained by using the 50,000 samples generated from the posterior distribution. Model fits, along with estimated credible regions, are illustrated in Figure 3 and Figure 4, where actual data points are represented by dotted lines and fitted values are denoted by solid lines. Both models exhibit strong fits, as indicated by the psuedo-$R^{2}$ values in Table 2. The rest of this section is organized as follows: Section 4.1 explores vaccination timing, hospital overload, and scenario analyses for both models. Section 4.2 investigates the similarity between fitted density plots using Hellinger distance in scenario analyses. The final subsection employs the Bayes factor, based on data from the State of Qatar, to compare the plausibility of Models (1) and (2). The implementation codes and detailed instructions can be found at https://github.com/elizabethamona/VaccinationTiming, accessed on 27 April 2025.

### 4.1. Scenario Analyses of Vaccine Efficacy and Timing of Hospital Overload Using Models 1 and 2

In response to the COVID-19 pandemic, the Qatari government implemented various interventions, the most stringent of which proved more effective in curtailing the rise of new COVID-19 cases compared to lenient interventions [5]. Here, we perform scenario analyses to assess the potential impact of not deploying the remaining stringent interventions. Specifically, we assess the number of days after which new COVID-19 cases would surpass the total hospital bed capacity of 3134 for COVID-19 patients in Qatar, thereby completely overwhelming the medical system. We also aim to analyze any variations in this outcome across six potential scenarios. The vaccine was made available on day 380 (31 December 2020) according to the available data, with an efficacy of 95% [37]. However, vaccine efficacy decreased as different variants emerged, leading us to adjust the vaccine efficacy to 94% in our analysis. The thresholds for early and late vaccinations were set to days 200 and 450, respectively, to provide insights into the vaccine’s effects after the first and second waves, respectively. Thus, the scenarios studied in this analysis consider 94% vaccine efficacy (scenario 1), 100% vaccine efficacy (scenario 2), early vaccination (day 200) with 94% vaccine efficacy (scenario 3), late vaccination (day 450) with 94% vaccine efficacy (scenario 4), early vaccination with 100% vaccine efficacy (scenario 5), and late vaccination with 100% vaccine efficacy (scenario 6). In each scenario, we calculate the posterior predictive distribution of the daily count of infected individuals, subsequently determining the range of number of days for which the number of infected individuals surpasses the predefined threshold of total available beds based on 50,000 posterior samples.

Findings from Model 1 reveal that under partial interventions, when vaccine efficacy was 94% (scenario 1), the number of days during which hospitals would have been overwhelmed ranged from 54 to 481 days. Vaccine efficacy of 100% (scenario 2) would have narrowed the range to 54–444 days, highlighting the substantial reduction in overwhelmed days with a fully efficacious vaccine, even under partial interventions. Early vaccine introduction (scenarios 3 and 5) did not significantly alter the overwhelmed period. However, delayed vaccination considerably widened the range to 57–487 days. In summary, Model 1 indicates that under partial interventions, the Qatari government’s provided hospital beds would never have sufficed without reaching critical capacity. Moreover, a 100% efficacious vaccine or early administration, irrespective of efficacy, would have resulted in fewer overwhelmed days compared to delayed vaccine administration.

Similar to the findings in Model 1, the analysis of Model 2 also revealed that under the scenario of 94% vaccine efficacy (scenario 1), hospitals would have been overwhelmed for a period of 55–453 days, while 100% vaccine efficacy (scenario 2) narrowed it to 53–443 days. The impact of early vaccine introduction under partial interventions was consistent across efficacy rates, with an overwhelmed period of 53–445 days under scenario 3 and 55–444 days under scenario 5. However, late vaccine introduction substantially extended the potential duration of hospital overload, ranging between 55 and 505 days under scenario 4 and between 53 and 513 days under scenario 6. Surprisingly, when applied late, vaccines with 100% efficacy (scenario 6) were found to be slightly less effective than vaccines with 94% efficacy (scenario 4), warranting further scientific investigation into the real-world dynamics on the pandemic.

### 4.2. Hellinger Distance Analysis

**Figure 5:**
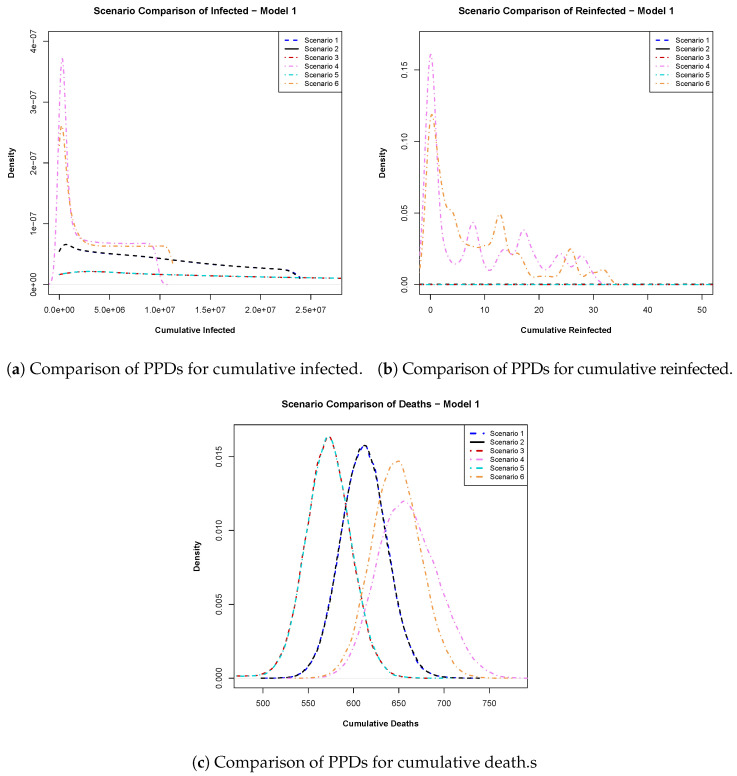
Plots of posterior predictive distributions (PPDs) of (**a**) cumulative infected, (**b**) cumulative reinfected, and (**c**) cumulative deaths at day 540 obtained from Model 1. These were generated using 50,000 samples from the posterior predictive distribution. Scenario 1 (94% vaccine efficacy) and scenario 2 (100% vaccine efficacy) represent the baseline timing of vaccine availability in Qatar (day 390). Scenario 3 (94%) and scenario 5 (100%) correspond to early vaccination (day 200), while scenario 4 (94%) and scenario 6 (100%) represent late vaccination (day 450).

**Figure 6:**
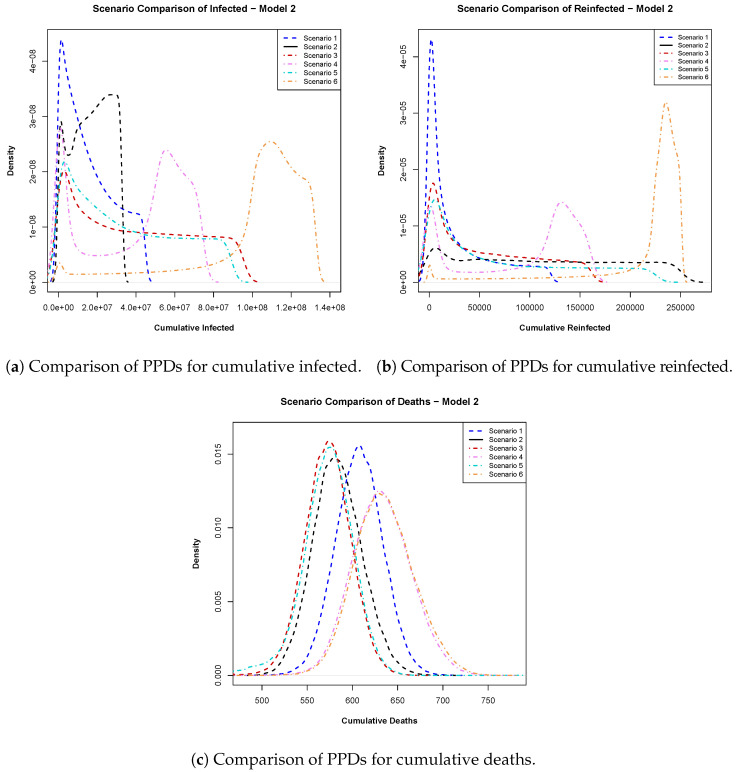
Plots of posterior predictive distributions (PPDs) of (**a**) cumulative infected, (**b**) cumulative reinfected, and (**c**) cumulative deaths at day 540 obtained from Model 2. These were generated using 50,000 samples from the posterior predictive distribution. Scenario 1 (94% vaccine efficacy) and scenario 2 (100% vaccine efficacy) represent the baseline timing of vaccine availability in Qatar (day 390). Scenario 3 (94%) and scenario 5 (100%) correspond to early vaccination (day 200), while Scenario 4 (94%) and Scenario 6 (100%) represent late vaccination (day 450).

Under each scenario, we also computed the posterior predictive distributions (PPDs) for cumulative infections, reinfections, and deaths at day 540, which is 10 days before the conclusion of our study period. These distributions were smoothed using kernel density smoothing (KDE) with a Gaussian kernel and default bandwidth. Plotting the smoothed PPDs allowed us to visualize the impact of varying vaccine efficacy and the timing of vaccine availability on infections, reinfections, and deaths since the onset of the pandemic and the introduction of vaccines in Qatar. Figure 5 displays the smoothed posterior predictive distributions for cumulative infections, reinfections, and deaths at day 540 from Model (1), while Figure 6 illustrates the same for Model (2). Subsequently, we conducted Hellinger distance analysis on these smoothed densities to estimate the ‘closeness’ of the different scenarios. Here, we summarize Figure 5 and Figure 6 and their Hellinger distance calculation findings for a similarity check between the six scenarios considered in the analysis. See Appendix A for detailed explanations, including the values of the similarity distance probabilities.

Model 1: In Figure 5a, we analyzed the posterior predictive distributions for cumulative infections calculated for day 540 within a 550-day time frame. Scenarios involving a 94% efficacious vaccine (scenario 1) and a 100% efficacious vaccine (scenario 2) exhibited high similarity, suggesting that pursuing an ideal vaccine did not significantly affect case control. Conversely, scenarios 1 and 3 (early vaccine with 94% efficacy) displayed substantial dissimilarity, emphasizing the impact of vaccination timing. Late vaccinations (scenarios 4 and 6) showed a preference for 100% vaccine efficacy over 94%.

Moving to Figure 5b and analyzing reinfections, we inferred that early vaccination remained preferable due to lower probabilities of excessively high reinfections in these scenarios. In Figure 5c, examining cumulative death density plots, early vaccination (scenarios 3 and 5) indicated fewer deaths than observed outcomes (scenarios 1 and 2). Late vaccinations (scenarios 4 and 6) indicated a higher cumulative death toll, with vaccine efficacy playing a role. These findings highlight the relationship between infections and deaths, mirroring real-world dynamics.

Model 2: In Figure 6a, the posterior predictive distributions for cumulative infections emphasize the importance of vaccine efficacy, favoring a 100% efficacious vaccine over a 94% efficacious vaccine, regardless of rollout timing. Figure 6b suggests that early vaccination remained preferable due to lower probabilities of excessively high reinfections. Finally, the cumulative PPDs for deaths in Figure 6c advocate for early vaccination with higher-efficacy vaccines, which was shown to result in fewer cumulative deaths.

### 4.3. Model Comparison Using Bayes Factor

We introduced two models in our study, each with distinct dynamics, aiming to identify the better explanatory model for observed data. Using the Bayes factor metric (detailed in Section 3.2), we calculated $BF_{1,2}$ as 50,013.35, which indicates that Model 1 is more probable and a better fit for the data than Model 2. This conclusion aligns with the higher pseudo-$R^{2}$ value of Model 1. Thus, Bayes factor analysis suggested that incorporating a secondary compartment ($S_{2}$) to accommodate individuals with recovered natural immunity and addressing the waning of vaccine-induced immunity with varied transition rates to the secondary susceptible compartment is more appropriate for representing the dynamics of the COVID-19 pandemic in the State of Qatar.

## 5. Discussion

In this paper, we explored the dynamics of infectious diseases through two distinct models, aiming to understand the impact of vaccination, reinfection, and interventions on disease courses based on real-life data. We conducted a comparative analysis using the Bayes factor, revealing that Model 1, considering varying susceptibilities post recovery, is significantly more probable than the simpler Model 2 based on COVID-19 data from Qatar. Our approach of employing the Bayes factor for model selection in the context of disease modeling is novel and, to the best of our knowledge, has not been explored before in the literature. Our research can also serve as a valuable tool for decision makers grappling with the formulation of challenging public health policies during a pandemic. It is evident from Figure 5a–c that, regardless of the vaccine’s effectiveness, getting vaccinated late is not recommended. Late vaccination increases the chances of more people becoming infected and reinfected and more deaths. The timing of vaccine rollout also matters; our results show that introducing vaccines earlier saved lives and reduced infections, although the overall decrease was not as significant as with early vaccination. Therefore, in the presence of partial interventions, early vaccination (scenarios 3 and 5) are the most effective in reducing active infections, reinfections, and deaths. If the vaccine had been introduced earlier, we could have saved more than the 50 lives, as mentioned in the study by Amona et al. [5], and the number of infectious individuals would have decreased significantly compared to the reduction observed when the vaccine was introduced much later. As a result, our research advocates for the implementation of early vaccination protocols in the event of a future pandemic.

A related study by LaJoie et al. [38] developed a data-calibrated, age-structured SEIR model to evaluate the combined impact of vaccination, waning immunity, and variant emergence across five countries. They found that earlier vaccination campaigns led to lower infection peaks and fewer cases, particularly in regions with delayed access to vaccines. While their model includes explicit compartments for waning immunity and booster doses, our study focuses on timing under simplified assumptions and arrives at a similar conclusion: early rollout reduces infections and deaths. A recent systematic review by Burch et al. [39] evaluated 47 age-structured transmission models and noted that most did not incorporate either reinfection or waning immunity, despite growing evidence of their relevance. Their call for models that better support long-term vaccine planning aligns with our approach, which integrates reinfection dynamics, vaccine timing, and uncertainty quantification to inform future pandemic strategies. Wang et al. [40] used a stochastic compartmental model to show that vaccination timing had a greater impact in terms of reducing COVID-19 mortality than either risk-based prioritization or the uptake rate. Although their analysis assumed long-lasting immunity and did not account for reinfection, their findings are consistent with our results—early vaccine rollout is critical to minimizing deaths. While our model incorporates vaccine-induced protection and reinfection dynamics, it does not stratify by age, comorbidity, or behavior, which limits its ability to reflect real-world heterogeneity in susceptibility and outcomes. We also did not simulate variant-specific transmission or antibody waning over time, as the available data did not support the direct modeling of immune decay. Instead, we captured reinfection through a structurally distinct secondary susceptible class, allowing us to study infection risk post recovery. In the absence of individual-level data, this framework still enables a robust evaluation of how vaccine timing and efficacy influence epidemic trajectories under reinfection risk.

To further emphasize the critical nature of timely vaccination, we conducted a sensitivity analysis exploring the repercussions of additional delays in vaccine deployment. Specifically, we examined the consequences of an additional one-month (30-day) delay and a one-and-a-half-month (45-day) delay in vaccination. Our findings reveal that, with these extended delays, the number of hospital overload daysremains a concern, consistently ranging from 55 to 505. This stable range persists, regardless of vaccine efficacy, underscoring the robustness of our results across different scenarios of delayed vaccination. Furthermore, when the vaccine is delayed for an extra month (implemented on day 480), we observe substantial increases of 7178 in COVID-19 cases and 62 in deaths. Although the reinfected population decreases by 30, this could be attributed to more initial infections leading to fatalities in those unable to recover due to delayed vaccination. Similar numbers of new cases and deaths are seen in the case of a 45-day delay in vaccination. This sensitivity analysis underscores the critical importance of timely vaccination, as delays not only escalate infections but also result in a higher mortality rate.

Our work was designed to investigate how reinfection dynamics and the type of immunity, whether vaccine-induced or post infection, help shape the course of an epidemic under different vaccination scenarios. One major contribution of our framework is its ability to differentiate between individuals susceptible to first-time infection and those susceptible to reinfection through a structurally distinct secondary susceptible compartment. We also separated vaccine-induced immunity from immunity following first infection, which allowed us to better capture the complexity of COVID-19 transmission—something often oversimplified in compartmental models that combine these effects. To allow for more granularity in how population subgroups and variant-specific effects are represented, future work may incorporate population heterogeneity and variant-specific immunity to support long-term vaccination strategies.

## Figures and Tables

**Table 1:** Initial conditions for Models (1) and (2).

Compartment	Initial Conditions	Source
$S\left(0\right)=S_{1}\left(0\right)$	2,695,122	Population of Qatar as of 30 December 2022
$S_{2}\left(0\right)$	2,695,122/6	Assumed
V(0)	0	From the data
E(0)	5	From [5]
I(0)	1	From the data
$R_{E}\left(0\right)=R_{I}\left(0\right)$	0	From the data
$I_{I}\left(0\right)$	0	From the data
$R_{R}\left(0\right)$	0	From the data
D(0)	0	From the data

**Table 2:** Estimated $S_{1}$EIRDV$S_{2}I_{I}R_{R}$ (Model 1) and SEIRDV$I_{I}R_{R}$ (Model 2) model parameters. These parameter values were estimated using the 50,000 posterior samples. Times are in days; rates are individuals per day, except $\alpha ,\zeta_{1}$, and $\zeta_{2}$, with rates in 10,000 individuals per day.

Model	Parameter	Description	Est. Mean	(0.025,0.975)	Psuedo-$R^{2}$
**Model 1**	$\alpha_{0}$	Transmission rate before intervention	0.00286	(0.00246,0.00306)	
	$\alpha_{1}$	Transmission rate after the first intervention	0.00084	(0.00081,0.00087)	
	$\alpha_{2}$	Transmission rate after the second intervention	0.00091	(0.00089,0.00095)	
	$\alpha_{3}$	Transmission rate after the third intervention	0.00076	(0.00075,0.00077)	
	$\alpha_{4}$	Transmission rate after the fourth intervention	0.00110	(0.00109,0.00111)	
	$\alpha_{5}$	Transmission rate after the fifth intervention	0.00089	(0.00088,0.00090)	
	$\alpha_{6}$	Transmission rate after the sixth intervention	0.00086	(0.00085,0.00088)	
	$\alpha_{7}$	Transmission rate after the seventh intervention	0.00097	(0.00097,0.00099)	
	$\alpha_{8}$	Transmission rate after the eighth intervention	0.00112	(0.00112,0.00113)	
	$\alpha_{9}$	Transmission rate after the ninth intervention	0.00132	(0.00246,0.00306)	
	β	Rate at which the exposed become infectious	0.06988	(0.06939,0.07075)	
	$\gamma_{10}$	Recovery rate from infections influenced by intervention	0.09097	(0.05473,0.10933)	
	$\gamma_{11}$	Recovery rate from infections influenced by intervention	0.13564	(0.13499,0.13883)	0.99176
	$\gamma_{12}$	Recovery rate from infections influenced by intervention	0.14758	(0.14192,0.14847)	
	$\gamma_{13}$	Recovery rate from infections influenced by intervention	0.11333	(0.11300,0.11370)	
	$\phi_{1}$	Reinfection rate influenced by intervention	0.00538	(0.00528,0.00543)	
	$\phi_{2}$	Reinfection rate influenced by intervention	0.01187	(0.01170,0.01244)	
	$\phi_{3}$	Reinfection rate influenced by intervention	0.02213	(0.02118,0.02312)	
	$\phi_{4}$	Reinfection rate influenced by intervention	0.00740	(0.00679,0.00805)	
	$\gamma_{2}$	Recovery rate from reinfections	0.14295	(0.14295,0.14295)	
	μ	Vaccination rate	0.00460	(0.00000,0.00920)	
	κ	Vaccine efficacy	0.94	(0.94,0.94)	
	η	Death rate	0.00021	(0.00021,0.00021)	
	$\zeta_{1}$	Waning rate of natural immunity	0.00202	(0.00001,0.00046)	
	$\zeta_{2}$	Waning rate of immunity due to vaccination	0.00119	(0.000170.00929)	
	$\alpha_{0}$	Transmission rate before intervention	0.00314	(0.00289,0.00329)	
	$\alpha_{1}$	Transmission rate after the first intervention	0.00089	(0.00084,0.00093)	
	$\alpha_{2}$	Transmission rate after the second intervention	0.00089	(0.00089,0.00090)	
	$\alpha_{3}$	Transmission rate after the third intervention	0.00077	(0.00076,0.00079)	
	$\alpha_{4}$	Transmission rate after the fourth intervention	0.00110,	(0.00109,0.00110)	
	$\alpha_{5}$	Transmission rate after the fifth intervention	0.00090	(0.00088,0.00091)	
	$\alpha_{6}$	Transmission rate after the sixth intervention	0.00087	(0.00086,0.00088)	
	$\alpha_{7}$	Transmission rate after the seventh intervention	0.00098	(0.00097,0.00099)	
	$\alpha_{8}$	Transmission rate after the eighth intervention	00114	(0.00113,0.00115)	
	$\alpha_{9}$	Transmission rate after the ninth intervention	0.00135	(0.00289,0.00329)	
	β	Infectious rate	0.07270	(0.06963,0.07616)	
	$\gamma_{10}$	Recovery rate from infections influenced by intervention	0.11527	(0.09406,0.12748)	
**Model 2**	$\gamma_{11}$	Recovery rate from infections influenced by intervention	0.13595	(0.13537,0.13651)	0.99160
	$\gamma_{12}$	Recovery rate from infections influenced by intervention	0.15138	(0.14984,0.15178)	
	$\gamma_{13}$	Recovery rate from infections influenced by intervention	0.11501	(0.11460,0.11544)	
	$\phi_{1}$	Reinfection rate influenced by intervention	0.01885	(0.01829,0.01945)	
	$\phi_{2}$	Reinfection rate influenced by intervention	0.01978	(0.01902,0.02071)	
	$\phi_{3}$	Reinfection rate influenced by intervention	0.02315	(0.02159,0.02480)	
	$\phi_{4}$	Reinfection rate influenced by intervention	0.01149	(0.01041,0.01260)	
	$\gamma_{2}$	Recovery rate from reinfections	0.14286	(0.14286,0.14286)	
	μ	Vaccination rate	0.00462	(0.0000,0.00925)	
	κ	Vaccine efficacy	0.94	(0.94,0.94)	
	η	Death rate	0.00021	(0.00021,0.00021)	
	$\zeta_{1}$	Waning rate of natural immunity	0.05374	(0.00090,0.10646)	
	$\zeta_{2}$	Waning rate of immunity due to vaccination	0.00359	(0.00000,0.00229)	

## Data Availability

The dataset and code used for this project can be found at https://github.com/elizabethamona/VaccinationTiming, accessed on 27 April 2025. The data include the daily cumulative number of confirmed infections, the cumulative number of recovered individuals, and the cumulative number of deaths for the State of Qatar starting on 28 February 2020. They also include the number daily reinfections and re-recovered individuals for Qatar starting from 23 June 2020 and the number of vaccinated individuals in the country from 31 December 2020. To ensure the reliability and robustness of our results, we conducted a comprehensive assessment of data quality. This involved thorough checks for consistency, accuracy, and adherence to epidemiological standards. Our collaboration with Dr. Abu-Raddad further ensured that the dataset met the highest standards of data integrity in the field.

## Appendix A. Details on Hellinger Distance Analysis

In our examination of Model 1 for posterior predictive distributions of cumulative infections at day 540 out of 550 in Figure 5a, scenarios involving the 94% efficacious vaccine (scenario 1) and the 100% efficacious vaccine (scenario 2) exhibited remarkably high similarity, as indicated by a low Hellinger Distance of 0.000020, indicating that the pursuit of an ideal vaccine may not have significantly influenced the effective control of cases within the system. Conversely, dissimilarity was substantial when comparing scenarios 1 and 3 (early vaccine with 94% efficacy), with a Hellinger distance of 0.340625. Late vaccinations (scenarios 4 and 6) resulted in more cumulative infections and demonstrated considerable dissimilarity, marked by a Hellinger distance of 0.034114, with 100% efficacy being more effective than 94% efficacy.

Shifting to reinfections, in the posterior predictive density plots from Model 1 in Figure 5b, scenarios 1 and 2 showed significant similarity (Hellinger distance: 0.000179), while dissimilarity was pronounced between scenarios 1 and 3 (Hellinger distance: 0.375517). Scenarios 3 and 5 displayed substantial similarity (Hellinger distance: 0.000012), but scenarios 4 and 6 showed some dissimilarity (Hellinger distance: 0.033001). The number of cumulative reinfections was also likely to be around 35 on day 540 when the vaccine was administered late (scenarios 4 and 6). The most substantial dissimilarity emerged when comparing scenarios 5 and 6, signified by a Hellinger distance of 0.531083.

The Model 1 posterior predictive cumulative death density plots in Figure 5c reveal that early vaccination (scenarios 3 and 5) would have resulted in a lower number of cumulative deaths compared to the observed outcomes (scenarios 1 and 2). Late vaccination (scenarios 4 and 6) would have produced a much higher cumulative death toll, and vaccine efficacy mattered in this case. Significant similarity was seen between scenarios 1 and 2 (Hellinger distance: 0.000091) and between scenarios 3 and 5 (Hellinger distance: 0.000278), while moderate and substantial dissimilarities were observed between scenarios 4 and 6 (Hellinger distance: 0.031157) and between scenarios 5 and 6 (Hellinger distance: 0.644322), respectively. These findings show that fewer infections lead to fewer deaths, while more infections lead to higher numbers of deaths, mimicking real-world dynamics.

In line with our analysis for Model 1, we computed Hellinger distances for the posterior predictive infected density plots in Model 2, as displayed in Figure 6a. Unlike Model 1, scenario 1 (94% efficacious vaccine) and scenario 2 (100% efficacious vaccine) demonstrated remarkable dissimilarity (Hellinger distance: 0.500488). Comparison of scenario 1 and scenario 3 (early vaccine with 94% efficacy) revealed a moderate degree of dissimilarity (Hellinger distance: 0.242202), and that of scenario 3 and scenario 5 (early vaccine at 100% efficacy) indicated moderate similarity as well (Hellinger distance: 0.013369). Conversely, the comparison between scenario 4 (late vaccine at 94% efficacy) and scenario 6 (late vaccine at 100% efficacy) revealed the most significant dissimilarity (Hellinger distance: 0.616759). The same trend was observed between scenarios 5 and 6 (Hellinger distance: 0.558894). Overall, the efficacy of the vaccine appeared to be crucial in Model 2, with a clear preference for a 100% efficacious vaccine over a 94% efficacious vaccine, irrespective of timing of the rollout.

Transitioning to reinfections, in the posterior probability density plots in Figure 6b, scenarios 1 and 2 exhibited substantial dissimilarity (Hellinger distance: 0.298233). Similarly, comparing scenarios 1 and 3, we observed substantial dissimilarity (Hellinger distance: 0.102579). Conversely, scenario 3 and scenario 5 displayed a lesser degree of dissimilarity (Hellinger distance: 0.07729328). Additionally, scenarios 4 and 6 were found to have significant dissimilarity (Hellinger distance: 0.5072856), while scenarios 5 and 6 were moderately dissimilar (Hellinger distance: 0.3656723). Thus, early vaccination was still preferred, as the probability of excessively high numbers of reinfections became improbable in these scenarios.

For the cumulative posterior predictive distributions of death in Figure 6c, our analysis indicated moderate similarity between scenarios 1 and 2 (Hellinger distance: 0.094946). Considerable similarity was also observed between scenario 3 and 5 (Hellinger distance: 0.006788) and scenarios 4 and 6 (Hellinger distance: 0.001163). The most significant dissimilarity was observed when comparing scenarios 5 and 6 (Hellinger distance: 0.440002). These results, once again, advocate for early vaccination, with the more efficacious vaccine resulting in fewer cumulative deaths.
